# Competition-related factors directly influence preferences for facial cues of dominance in allies

**DOI:** 10.1007/s00265-016-2211-2

**Published:** 2016-10-05

**Authors:** Christopher D. Watkins, Benedict C. Jones

**Affiliations:** 1 Division of Psychology, School of Social and Health Sciences, Abertay University, Dundee, Scotland DD1 1HG UK; 2 Face Research Laboratory, Institute of Neuroscience and Psychology, University of Glasgow, 58 Hillhead Street, Glasgow, G12 8QB UK

**Keywords:** Social brain hypothesis, Alliances, Politics, Sex differences, Within-sex competition

## Abstract

**Abstract:**

Alliance formation is a critical dimension of social intelligence in political, social and biological systems. As some allies may provide greater “leverage” than others during social conflict, the cognitive architecture that supports alliance formation in humans may be shaped by recent experience, for example in light of the outcomes of violent or non-violent forms intrasexual competition. Here we used experimental priming techniques to explore this issue. Consistent with our predictions, while men’s preferences for dominant allies *strengthened* following losses (compared to victories) in violent intrasexual contests, women’s preferences for dominant allies *weakened* following losses (compared to victories) in violent intrasexual contests. Our findings suggest that while men may prefer dominant (i.e. masculine) allies following losses in violent confrontation in order to facilitate successful resource competition, women may “tend and befriend” following this scenario and seek support from prosocial (i.e. feminine) allies and/or avoid the potential costs of dominant allies as long-term social partners. Moreover, they demonstrate facultative responses to signals related to dominance in allies, which may shape sex differences in sociality in light of recent experience and suggest that intrasexual selection has shaped social intelligence in humans.

**Significance statement:**

Although alliance formation is an important facet of social intelligence in political and biological systems, we know relatively little about the cognitive processes involved in social preferences for allies. As recent experience may alter the leverage provided by different social partners, here we tested whether preferences for facial cues to dominance-prosociality (masculinity-femininity) alter in light of recent experience of violent and economic contests for status. Our findings demonstrate sex-specific responses to these facial cues. While men’s preferences for facial cues related to dominance in allies *strengthen* following losses (compared to wins) in violent contests, women’s preferences for facial cues related to dominance in allies *weaken* following losses (compared to wins) in violent contests. These findings suggest that intrasexual selection, in part, has shaped the evolution of social intelligence in humans as revealed in flexibility in social preferences for allies.

## Introduction

An important aspect of social intelligence is the ability to cooperate within strategic alliances in order to maximise reproductive fitness (see DeScioli and Kurzban 2009 for discussion). Many non-human species form both coalitions, where two parties simultaneously aggress against a third party, and longer-term alliances, where coalitions are revisited over time, normally against multiple opponents (see Harcourt and de Waal 1992). For example, male dolphins form both small and stable, and larger, flexible alliances with other males who, in turn, enhance their ability to compete for access to mates (e.g. Connor et al. 1999, 2001; Whitehead and Connor 2005). Moreover, male wild Guinea baboons form different levels of alliance with other males (Patzelt et al. 2014) and, in some primates, male alliances directly increase their reproductive success and dominance rank (Schülke et al. 2010; Gilby et al. 2013). In other examples, such as Camargue horses (Feh 1999) and male chimpanzees (Duffy et al. 2007), support from high-ranking partners facilitates access to mates, and, among male savannah baboons, coalitions improve fighting ability against rivals (Noë and Sluijter 1995; see also Caro and Collins 1987 and Packer and Pusey 1982 for coalitions in male cheetahs and lions). Moreover, white-faced capuchins form coalitions based on both shared affinity and the partner’s rank exceeding that of a rival (Perry et al. 2004), and ravens provide support to partners according to affinity (e.g. indexed via grooming) and the partner’s dominance rank in order to gain future agonistic support (Fraser and Bugnyar 2012). Female savannah baboons also provide aid to females in disputes (Silk et al. 2004), with longevity increasing among females, independent of dominance rank, with the provision of close social bonds (e.g. as indexed by frequent grooming and/or contact within a set time period; Silk et al. 2010). Collectively, there are potential benefits to alliances with conspecifics, including the facilitation of successful resource competition.

Humans form large and complex social networks (e.g. Hill and Dunbar 2003; Snyder 2007), with high quality social and emotional support from others having direct effects on proxies for reproductive fitness such as health indexed via longevity (see Holt-Lunstad et al. 2010 for a meta-analytic review), which, in turn, can maximise reproductive fitness over generations (e.g. raising offspring to independence; see Lawson and Mace 2011 for general discussion). Cooperative alliances may have been important for status acquisition throughout human evolution, as individuals share valued traits and expertise with one another rather than inflicting costs on them for direct access to resources, providing “leverage” (Hand 1986) during social conflict (see Henrich and Gil-White 2001). Consistent with this proposal, fossil record evidence suggests that violent male-male competition was an important factor in the evolution of human cooperation (Bowles 2009) and physical traits that denote formidability (i.e. ability to dominate in a contest) appear to be valued in leaders, in part, in order to attract group members who can resolve social conflicts more generally (i.e. also on a smaller-scale; see van Vugt and Grabo 2015 for recent discussion). Indeed, *both* physically dominant and prestigious men have higher fertility, more support from allies and are more likely to be deferred to by competitors (von Rueden et al. 2011), and male coalitionary aggression facilitates reproductive opportunities for males and community cohesion (Macfarlan et al. 2014). Collectively, both direct (i.e. violent) and indirect competition for resources (acquiring resources through force versus consumption and/or skill respectively, e.g. Smallegange et al. 2006) may have shaped the cognitive architecture that underpins alliance formation in humans (see also DeScioli and Kurzban 2009 for discussion).

Sexually dimorphic physical characteristics signal traits that may be sought after in allies, as they play an important role in within-sex competition (reviewed in Emlen 2008; Santos et al. 2011) and are correlated with male dominance rank (e.g. Pelletier and Festa-Bianchet 2006), fighting ability (e.g. Bergeron et al. 2010), physical strength (e.g. Malo et al. 2009) and reproductive fitness (e.g. Preston et al. 2003) in many non-human animal species. In humans, although other cues indicate a social partner’s relative dominance, such as eye gaze (Jones et al. 2010) and anger (Ackerman et al. 2006), masculine physical characteristics, such as low voice pitch, high facial width to height ratio and muscularity, are positively associated with both perceptions of dominance and measures of actual traits related to dominance (e.g. Stirrat and Perrett 2010; Petersen et al. 2013; reviewed in Puts 2010; Watkins et al. 2010a) and feminine physical characteristics, such as softer face shape and large eyes, are associated with prosocial traits such as perceived ability to provide high-quality social support (see Watkins et al. 2012a for discussion in the context of emotional support and investment). Indeed, the effects of digitally enhanced facial masculinity on dominance perceptions are substantial (see Puts 2010 for a summary) and the speed of trait judgements of faces is functionally adaptive if fast approach/avoid behaviour on these dimensions is favoured over accurate approach/avoid behaviour on identical dimensions (reviewed in Todorov et al. 2008). Collectively, in light of the benefits of avoiding costly intrasexual conflict (Puts 2010) and in selecting cooperative social partners (Queller 2011), sexually dimorphic characteristics may be utilised at minimal acquaintance in order to approach or avoid potential social partners.

While dominance and prosociality are potentially valuable traits in allies and are gauged, in part, from sexually dimorphic facial characteristics (Puts 2010; Watkins et al. 2012a), preferences for cues to these traits may be facultative and respond in light of recent experience such as one’s own success or failure in contests for status. Contest outcomes moderate engagement in further confrontation in various species (reviewed in Hsu et al. 2006). In humans, men’s perceptions of other men’s dominance alter in light of contest outcomes, such that losers of confrontations perceive facial cues of dominance (i.e. facial masculinity) to be more salient than winners do (i.e. losers are more likely to associate facial masculinity with *high* dominance; Watkins and Jones 2012; see also Welling et al. 2016 for further discussion of experience and competition). Recent work, however, suggests that having an ally decreases judgements of formidability in potential rivals (Fessler and Holbrook 2013), complementing work which demonstrates that support from coalition partners predicts success in dyadic conflict (von Rueden et al. 2008). Social perceptions of allies may therefore function to increase the leverage of individuals (Hand 1986) when attempting to resolve social conflicts in light of recent experience (see also DeScioli and Kurzban 2009).

Following on from priming experiments that test for effects of recent confrontations on judgements of potential rivals (Watkins and Jones 2012), here we adapt this paradigm to test for effects of recent contests for status on judgements of potential allies. Specifically, we test whether the nature of competition within the environment (direct/violent versus indirect/economic) and the outcomes of recent contests (win or loss) moderate preferences for sexually dimorphic facial characteristics in allies. We predict that sexually dimorphic cues will be preferred, on average, when men, but not women, judge other men as allies, in light of the greater fitness advantages to males who formed large groups to facilitate successful resource competition against rival groups (Bowles 2009; Benenson et al. 2013), and as men with dominance-related characteristics are better-placed to provide leverage to individuals as allies via the threat they pose to those groups (i.e. “parochial altruism”; Choi and Bowles 2007; McDonald et al. 2012).

Secondly, by manipulating masculine shape cues in faces, we predict sex-specific responses to facial cues related to dominance in allies following different outcomes and forms of resource competition. Whereas male sociality is oriented toward behaviours that facilitate successful competition for mates and/or resources (e.g. seeking *instrumental* support; McDonald et al. 2012; Benenson et al. 2013), female sociality is characterised by behaviours that maximise their and/or their offspring’s personal safety, for example by recruiting allies who provide social and *emotional* support or physical protection, particularly in response to stressful circumstances (i.e. “tending and befriending”; Taylor et al. 2000). Indeed, recent experimental evidence suggests that facial cues to threat are more salient to men in contexts where violent male-male competition is likely to be intense (i.e. to maximise success in competition) but are more salient to women in contexts where self-protection is of greater concern (i.e. to avoid further threats; Watkins et al. 2013). Although prior research suggests that there are no sex differences in preferences for allies with a competitive advantage when the likelihood of winning is manipulated in economic games (Benenson et al. 2009), this does not rule out the possibility that preferences for facial masculinity-femininity in allies will vary differently for women versus men in light of recent experience of competition, especially as violent within-sex competition has lower benefits and greater costs for women more generally (reviewed in Archer 2009; Campbell 2013). Thus, we predict that recent experience (win or loss) of direct versus indirect competition will have different effects on how men judge allies (to facilitate successful competition) compared to how women judge allies (to seek emotional support and/or protection). While stronger preferences among men for *dominant* allies following losses in violent status contests (compared to wins) would function, in part, to recruit allies who are better placed to facilitate successful competition (i.e. parochial altruism; Choi and Bowles 2007; McDonald et al. 2012) and increase dominance rank (e.g. in primates; Schülke et al. 2010; Gilby et al. 2013; in humans; von Rueden et al. 2008, 2014), stronger preferences among women for *prosocial* allies following losses in violent contests for status (compared to wins) would function to recruit emotionally supportive (i.e. investing) partners (reviewed in Watkins et al. 2012a) and/or avoid dominance-related (i.e. low investing) characteristics in social partners when self-protection is at a premium. This prediction is particularly relevant to close female bonds (Taylor et al. 2000) if unsupportive close bonds are poor solutions to dealing with stressful circumstances (see Taylor 2006 for discussion) and proxies for these traits such as emotional coldness are gauged from masculine facial characteristics (Perrett et al. 1998). Moreover, this predicted pattern of findings is likely to be specific to when competition is direct (violent) as opposed to indirect (economic). For example as lower income *relative* to one’s peers negatively predicts measures of life satisfaction (Boyce et al. 2010) and as prior work suggests that attraction to facial femininity is greater when concerns about resource scarcity are salient (Little et al. 2007; Lee and Zietsch 2011), prosocial allies may be preferred when economic competition is intense such as when competing against peers for promotion, particularly in adverse circumstances where the costs of preferring dominance-related characteristics in social partners are greater, in light of, for example their low egalitarianism (e.g. Stirrat and Perrett 2010; Petersen et al. 2013). Thus, recent experience of economic competition may shape ally preference such that apparent dominance is avoided in allies (i.e. weaker preferences for facial masculinity) and/or prosocial individuals are sought as allies (i.e. stronger preferences for facial femininity).

## Method

### Participants

Two hundred forty-six participants (121 men, mean age = 22.75 years, SD = 6.03 years) completed the experiment online. Participants were recruited from links on social bookmarking sites, such as stumble upon. Previous research on social perceptions of faces has demonstrated that laboratory and online studies produce equivalent results (e.g. Watkins et al. 2010a, 2012b) and are comparable more generally (Gosling et al. 2004). Responses from duplicate IP addresses were not recorded. As the experiment was run online, recording of responses to the task is free from experimenter bias (i.e. as such, blinded methods were used when behavioural data were recorded).

### Stimuli

**Fig. 1:**
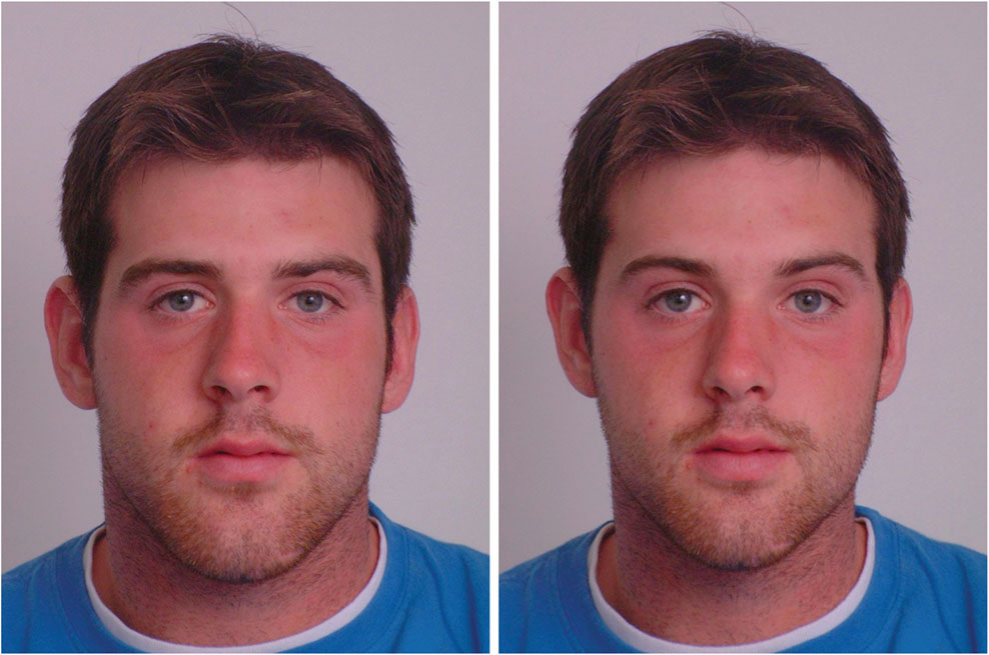
Examples of masculinised (*left*) and feminised (*right*) versions of face images used in our experiment

Following previous studies of perceptions of masculinised versus feminised faces (e.g. Perrett et al. 1998; Jones et al. 2010; Watkins et al. 2010a, b), we used prototype-based image transformation to objectively and systematically manipulate sexually dimorphic aspects of 2D shape in digital face images. Following these studies, 50 % of the linear differences in 2D shape between symmetrised versions of a male and female prototype were added to or subtracted from digital face images of 20 young white adult men (*M*_age_ = 19.5 years, SD = 2.3 years) and 20 young white adult women (*M*_age_ = 18.4 years, SD = 0.7 years; see Tiddeman et al. 2001 for further technical details). The faces used here were of Canadian students and have been used in previous work on social perceptions of dominance (e.g. Watkins et al. 2010a, 2012b). The resultant masculinised and feminised versions of the individual faces images differ in sexually dimorphic aspects of 2D shape but are identical in other regards (e.g. identity, symmetry, skin colour and texture; Rowland and Perrett 1995). Examples of masculinised and feminised face images are shown in Fig. 1.

This process created 20 pairs of male face images and 20 pairs of female face images in total, with each pair consisting of a masculinised and feminised version of the same individual. These methods affect perceptions of dominance, physical strength and masculinity in the predicted manner (e.g. DeBruine et al. 2006; Jones et al. 2010). Masculinised versions of men’s and women’s faces are perceived as more dominant than feminised versions of men’s and women’s faces (e.g. Watkins et al. 2010a, 2012b).

### Procedure

The experiment consisted of two phases: an initial priming phase and, subsequently, an ally preference test (adapted from Watkins and Jones 2012; Watkins et al. 2012a). In the initial priming phase of the experiment, each participant was randomly allocated to one of four conditions: a condition where they were instructed to imagine winning a physical fight, a condition where they were instructed to imagine losing a physical fight, a condition where they were instructed to imagine winning a contest for promotion and a condition where they were instructed to imagine losing a contest for promotion. Contests for promotion against same-sex colleagues have been used as scenarios in prior research on behavioural responses to competition (Griskevicius et al. 2009). The participants were given the following instructions: “Please take a moment to imagine that you have just been involved in a ‘physical fight’/‘contest for promotion’ with ‘someone’/‘a colleague’ of the same sex and age as you and that you ‘won’/‘lost’ the ‘fight’/‘contest’. Imagine how ‘winning the fight’/‘winning the contest’ made you feel/losing ‘the fight’/‘out on promotion’ made you feel and visualize yourself ‘winning’/‘losing’ ‘the fight’/‘being told that you were favoured over your colleague’/‘your colleague was favoured over you’”. Participants were allocated to one experimental scenario only. Before moving on to the ally preference test, participants rated how vividly they had imagined the scenario on a 1 (not very vivid) to 7 (very vivid) scale. Participants can accurately rate the vividness of their mental imagery (Pearson et al. 2011).

Immediately after the priming phase of the experiment, the participants completed an ally preference test. In this test, the participants were shown 20 pairs of male faces and 20 pairs of female faces, with each pair consisting of a masculinised and feminised version of the same individual. On each trial, the participants were asked to indicate which face in each pair they thought would make the better ally, and how much more suitable that individual would be as an ally. The participants were asked to choose the better ally and indicate their preference for the chosen face (relative to the other face in the pair) using the response options “much better ally”, “better ally”, “somewhat better ally” and “slightly better ally”. Trial order, the sex of face and the side of the screen on which any given image (masculinised or feminised) was shown were all fully randomised.

### Initial processing of data

Following prior work on systematic variation in judgements of masculinised versus feminised versions of faces (e.g. Watkins et al. 2010b), responses were coded using the following scale:

1. 0–3: feminised face rated a much better ally (=0), better ally (=1), somewhat better ally (=2) or slightly better ally (=3) than the masculinised face.
2. 4–7: masculinised face rated a slightly better ally (=4), somewhat better ally (=5), better ally (=6) or much better ally (=7) than the feminised face.

We used this data to calculate a participant’s average score on the ally preference test, separately for judgements of men’s faces and judgements of women’s faces. Higher values indicate a stronger tendency to perceive masculine individuals as better allies than feminine individuals. Use of np2 in analyses indicates the effect size measure partial eta squared.

## Results

First we carried out initial one-sample *t* tests to compare overall preferences for masculinity-femininity in allies with what would be expected by chance alone (i.e. 3.5). These analyses revealed that while participants tended to perceive masculinised versions of men’s faces to be better allies than feminised versions of men’s faces (*M* = 3.61, 95 % CI [−0.00, 0.23]; *t*(245) = 1.94; *p* = 0.054, *r* = 0.12), they perceived feminised versions of women’s faces to be better allies than masculinised versions of women’s faces (*M* = 3.22, 95 % CI [−0.38, −0.17]; *t*(245) = 5.20; *p* < 0.001, *r* = 0.32). Analysing the data separately for male and female judges revealed that men preferred masculine men more than feminine men as allies (*M* = 3.73, 95 % CI [0.06, 0.40]; *t*(121) = 2.64; *p* < 0.01, *r* = 0.23), but women had no overall preference for masculine or feminine men as allies (*M* = 3.50, 95 % CI [−0.15, 0.16]; *t*(125) = 0.01; *p* = 0.99). Both men (*M* = 3.24, 95 % CI [−0.42, −0.11]; *t*(121) = 3.33; *p* < 0.01, *r* = 0.29) and women (*M* = 3.21, 95 % CI [−0.43, −0.15]; *t*(125) = 4.04; *p* < 0.01, *r* = 0.34) preferred feminine women more than masculine women as allies.

Next, we carried out a mixed design ANOVA with scores on the ally preference test as the dependent variable; the within-subject factor was *face sex* (men’s faces, women’s faces) and the between subjects factors were *contest type* (physical fight, promotion), *contest outcome* (win, loss) and *sex of participant* (male, female). These analyses revealed a significant main effect of *face sex* (*F*(1, 238) = 65.17; *p* < 0.001, np2 = 0.22), whereby participants were more likely to choose masculine allies when judging men’s faces (*M* = 3.61, SEM = 0.06) than women’s faces (*M* = 3.22, SEM = 0.05). This main effect was qualified by an interaction with *contest type* (*F*(1, 238) = 6.78; *p* = 0.01, np2 = 0.03), however. This interaction indicated that the tendency to prefer masculinity more in allies when judging men than when judging women was significantly greater when judging allies after physical fights (*M*_male_ = 3.96, SEM = 0.09, *M*_female_ = 3.44, SEM = 0.08, *r* = 0.34) than when judging allies after contests for promotion (*M*_male_ = 3.25, SEM = 0.06, *M*_female_ = 3.00, SEM = 0.07, *r* = 0.14). We also observed a significant interaction between *face sex* and *sex of participant* (*F*(1, 238) = 6.04; *p* = 0.015, np2 = 0.03). This interaction demonstrated that the tendency to prefer masculine allies more when judging men than women was greater in male participants (*r* = 0.27) than it was in female participants (*r* = 0.21).

**Fig. 2:**
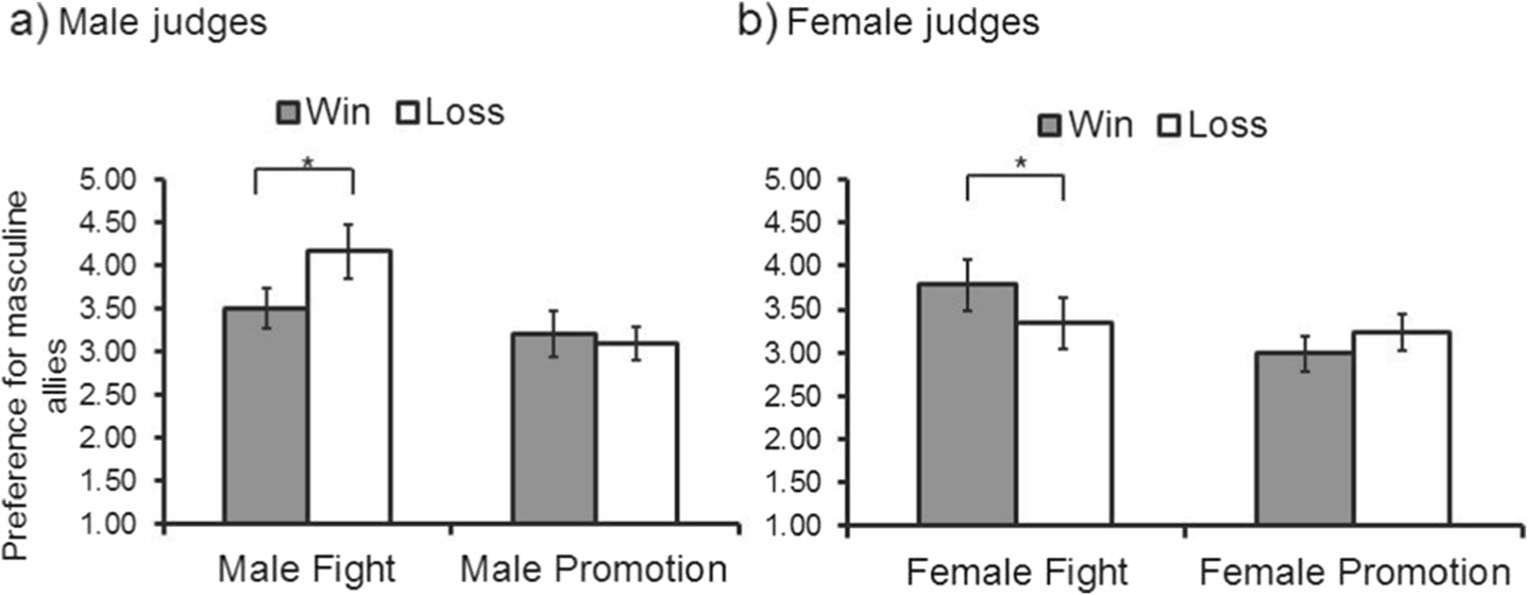
The significant interaction between *contest type*, *contest outcome* and *participant sex* on preferences for facial cues related to dominance in allies (*p* < 0.001, np2 = 0.06). While men’s preference for dominance-related characteristics in allies are *stronger* after a loss compared to a win in a violent contest for status (*r* = 0.42; **a**), women’s preference for dominance-related characteristics in allies are *weaker* after a loss compared to a win in a violent contest for status (*r* = 0.25; **b**). *Error bars* show 95 % confidence intervals

We also observed a significant effect of *contest type* (*F*(1, 238) = 39.27; *p* < 0.001, np2 = 0.14). This effect reflected a stronger preference for masculine allies following a physical fight (*M* = 3.70, SEM = 0.08) than following a contest for promotion (*M* = 3.12, SEM = 0.05). A significant interaction between *contest outcome* and *participant sex* (*F*(1, 238) = 4.22; *p* < 0.05, np2 = 0.02) indicated that men’s preference for masculine allies was stronger after a loss than a win in a contest for status while women’s preference for masculine allies was weaker after a loss than a win in a contest for status (both *t*s < 1.60, both *p*s > 0.11). Of central interest to our hypotheses, we observed a significant three-way interaction between *contest type*, *contest outcome* and *participant sex* (*F*(1, 238) = 16.14; *p* < 0.001, np2 = 0.06; see Fig. 2). No other significant effects or interactions were found (all *F*s < 3.13, all *p*s > 0.07).

In order to interpret our significant three-way interaction between *contest type*, *contest outcome* and *participant sex*, we conducted separate ANOVAs for men and women, collapsed across sex of face. This analysis revealed that *contest type* (*F*(1, 117) = 29.98; *p* < 0.01, np2 = 0.20) and *contest outcome* (*F*(1, 117) = 4.90; *p* = 0.03, np2 = 0.04), as well as an interaction between *contest type* and *contest outcome* (*F*(1, 117) = 9.12; *p* < 0.01, np2 = 0.07) had significant effects on men’s preference for facial cues to dominance in allies. Independent samples *t* tests revealed that while men’s preferences for facial cues to dominance in allies was stronger following a loss (*M* = 4.16, SEM = 0.16) compared to a win (*M* = 3.51, SEM = 0.11) in a physical fight (*t*(52.63) = 3.38, *p* < 0.01, *r* = 0.42, 95 % CI [0.27, 1.05]), they did not alter according to the outcomes of a contest for promotion (*t*(59) = 0.64, *p* = 0.53, *r* = 0.08, 95 % CI [−0.42, 0.22]).

When analysing women’s data, there was a significant effect of *contest type* (*F*(1, 121) = 11.70; *p* < 0.01, np2 = 0.09) but not *contest outcome* (*F*(1, 121) = 0.51; *p* = 0.48) on their preference for facial cues to dominance in allies. The interaction between *contest type* and *contest outcome* was significant, however (*F*(1, 121) = 7.12; *p* < 0.01, np2 = 0.06). Women’s preferences for facial cues to dominance in allies were weaker following a loss (*M* = 3.34, SEM = 0.14) compared to a win (*M* = 3.78, SEM = 0.15) in a physical fight (*t*(65) = 2.12; *p* = 0.04, *r* = .25, 95 % CI [−0.85, −0.03]) but tended to be stronger following a loss (*M* = 3.24, SEM = 0.10) compared to a win (*M* = 2.99, SEM = 0.10) in a contest for promotion (*t*(56) = 1.78; *p* = 0.08, *r* = 0.23, 95 % CI [−0.03, 0.54]).

Follow-up one-sample *t* tests against chance (i.e. 3.5) for each of our experimental conditions (separated by sex of participant) demonstrated that men preferred masculine allies after a loss in a physical fight (*M* = 4.16, 95 % CI [0.34, 0.99]; *t*(29) = 4.19; *p* < 0.001, *r* = 0.36) and preferred feminine allies after both a loss (*M* = 3.10, 95 % CI [−0.60, −0.21]; *t*(34) = 4.15; *p* < 0.001, *r* = 0.33) and win in a contest for promotion (*M* = 3.20, 95 % CI [−0.57, −0.04]; *t*(25) = 2.33; *p* = 0.03, *r* = 0.22). Women preferred feminine allies after both a loss (*M* = 3.24, 95 % CI [−0.47, −0.05]; *t*(25) = 2.52; *p* = 0.02, *r* = 0.24) and a win in a contest for promotion (*M* = 2.99, 95 % CI [−0.71, −0.31]; *t*(31) = 5.21; *p* < 0.001, *r* = 0.42). In our remaining priming conditions, men or women did not prefer masculine-feminine allies at levels that differed from chance (all *t*s < 1.91, all *p*s > 0.07). Of note, even where preferences for masculine-feminine allies do not differ from chance, and of central interest to our hypotheses, our higher order three-way interaction demonstrates that men’s social responses to allies differ significantly from women’s social responses to allies. Moreover, our separate ANOVAs for men and women demonstrate relative strengthening/weakening of preferences for facial masculinity-femininity in allies according to our primed experimental contexts.

A separate ANOVA on the dependent variable rated vividness of imagery, with the between-subject factors *contest type*, *contest outcome* and *participant sex*, confirmed that the rated vividness of imagery was equivalent across our four scenarios and that men and women did not differ from one another in their rated vividness of mental imagery across these scenarios (all *F*s < 1.97, all *p*s > 0.16). Rerunning all analyses with participant age entered as a covariate in the model did not alter any of our results.

## Discussion

Here we report novel evidence that sexually dimorphic characteristics are used to assess individuals as potential allies. Firstly, both men and women, on average and across contexts, associated feminine shape cues in other women with their suitability as an ally. In contrast, and consistent with our initial predictions, while men associated masculine shape cues in other men with suitability as an ally, women had no overall preference for masculine or feminine men as allies. As masculine (i.e. dominant looking; Puts 2010) men are better-placed to provide leverage as allies via the threat they pose to external groups (i.e. parochial altruism; Choi and Bowles 2007; McDonald et al. 2012; von Rueden et al. 2014), these initial findings are consistent with our prediction that men’s preference for masculine allies functions, in part, to improve dominance rank. Preference for dominant-looking (i.e. masculine) allies may, in turn, facilitate successful competition against rival groups, an important concern for males over evolutionary history (Bowles 2009; Benenson et al. 2013; see also van Vugt and Grabo 2015 for discussion of dominance and leadership in general contexts).

Additionally, our priming experiment suggests facultative responses to facial cues related to dominance in light of the (i) means and (ii) outcomes of recent status contests. Our significant three-way interaction suggests sex-specific preferences for cues to dominance (i.e. masculine faces) in light of recent experience of intrasexual competition. While men’s preference for dominance-related characteristics in allies was stronger following losses (compared to wins) in violent contests for status, women’s preference for dominance-related characteristics in allies was weaker following losses (compared to wins) in violent contests for status. These findings are consistent with the proposal that while men would choose allies in light of recent losses in order to provide leverage and facilitate successful competition (e.g. Schülke et al. 2010), women would choose allies in light of recent losses in order to seek prosocial/supportive allies (Watkins et al. 2012a) and/or to avoid formidable allies following aggressive conflict, where the costs to pursuing such relationships may be particularly substantial. While research has explored context-dependent judgements of cues related to dominance in romantic (reviewed in Little et al. 2011) and competitive interactions (see Fessler and Holbrook 2013 for recent discussion), this work provides novel evidence for contextual factors that shape associate choice. The facultative responses reported here may play an important role in human sociality when confronted by new environments or groups.

Although our higher-order interaction demonstrates that the outcomes of different forms of status competition have different effects on men’s versus women’s preferences for traits related to dominance in allies, post hoc tests suggest that the effects of recent experience of economic competition (competing for promotion) on preferences for traits related to dominance in allies are less clear. These tests suggest that women tend to strengthen their preference for dominance-related characteristics in allies after failing in a contest for promotion against a same-sex peer (compared to succeeding). While the effects of our “minimal manipulation” may well be substantial when tested in the field (Prentice and Miller 1992), future work could test for effects of recent experience on ally choice in organisations or groups, using complementary methods such as behavioural observation or experimental war games (see Johnson et al. 2006). It is worth noting however that our experimental manipulation suggests that minimal cues are sufficient to moderate ally choice, demonstrating great flexibility in the cognitive architecture that underpins sociality in humans.

Potential limitations of our work require further discussion. For example in non-humans, *coalitions*, where two conspecifics simultaneously aggress against a third party, differ from *alliances*, where coalitions are revisited and reformed over time, usually against multiple opponents (Harcourt and de Waal 1992). Here, our participants were asked to judge others for their suitability as potential allies from face cues alone. While our data do not speak directly to the time course of this phenomena in humans (i.e. short-term stable alliances versus longer-term repeated alliances), our findings demonstrate that short-term changes to the nature of the environment alter social judgements of allies. Further work on responses to allies in the short- versus long-term would likely prove fruitful, particularly in light of the multi-level complexity of human alliance politics over time and space (Snyder 2007) and the functional basis of episodic memory in humans for simulating future outcomes based on past transactions (see Suddendorf and Corballis 2007 for discussion). Secondly, although the results from our minimal manipulation, whereby we simply ask people to imagine themselves in different scenarios, suggest that similar effects in the real world may be substantial (Prentice and Miller 1992), further work could potentially test for behavioural responses to potential allies following actual contest outcomes, for example among professional fighters, sportspeople or work colleagues. Finally, paradigms other than the forced choice paradigm used here may also be fruitful for investigating the decision-making processes involved in ally choice, such as observation of same-sex group behaviour within the laboratory or field.

In summary, we used experimental priming techniques to demonstrate that competition-related factors have direct effects on the “trade-off” between preferring dominant versus prosocial characteristics in allies and that these effects differ for men’s versus women’s judgements of allies in ways that can be understood in light of research on sex differences in primate sociality (e.g. Bowles 2009; Benenson et al. 2013). Theoretical perspectives suggest that an important factor in the evolution of the social brain, or “Machiavellian intelligence” (see Dunbar and Shultz 2007) was the increasing threat posed by competition for scarce resources as humans began to master their surrounding environment (Flinn et al. 2005). Our findings provide direct experimental evidence that intrasexual competition may have been an important factor in shaping the cognitive architecture that underpins alliance formation, a key facet of social intelligence in humans.
